# Anticorrosion and Antimicrobial Evaluation of Sol-Gel Hybrid Coatings Containing *Clitoria ternatea* Modified Clay

**DOI:** 10.3390/gels9060490

**Published:** 2023-06-14

**Authors:** Milad Sheydaei, Milad Edraki, Seyyed Mehdi Radeghi Mehrjou

**Affiliations:** 1 Faculty of Polymer Engineering, Sahand University of Technology, Tabriz P.O. Box 51335-1996, Iran; 2 Polymer Department, Technical Faculty, South Tehran Branch, Islamic Azad University, Tehran P.O. Box 19585-466, Iran; 3 Department of Civil and Architecture Engineering, Technical and Vocational University of Iran [Guilan, Rasht (Chamran)], Tehran 1435661137, Iran

**Keywords:** sol-gel, *Clitoria ternatea*, coating, anticorrosion, antimicrobial

## Abstract

In this study, *Clitoria ternatea* (CT) was incorporated into the structure of sodium montmorillonite (Na^+^-MMT), then these new nanoparticles (CT-MMT) were added to sol-gel-based hybrid silanol coatings (SGC). The results of the CT-MMT investigation using Fourier transform infrared spectroscopy (FTIR), X-ray diffraction (XRD), scanning electron microscopy (SEM), and transmission electron microscope (TEM) confirmed the presence of CT in the structure. The results of polarization and electrochemical impedance spectroscopy (EIS) tests showed that the presence of CT-MMT in the matrix improves corrosion resistance. The EIS results showed that the coating resistance (R_f_) of the sample containing 3 wt.% CT-MMT after immersion was 687 Ω·cm^2^, while this value was 218 Ω·cm^2^ for pure coating. CT and MMT compounds improve corrosion resistance by blocking anodic and cathodic regions, respectively. Additionally, the presence of CT in the structure created antimicrobial properties. CT contains phenolic compounds that have the ability to suppress by membrane perturbation, reduction of host ligands adhesion, and neutralizing bacterial toxins. Therefore, CT-MMT showed inhibitory effects and killing of *Staphylococcus aureus* (gram-positive bacteria) and *Salmonella paratyphi-A serotype* (gram-negative bacteria), and also improved corrosion resistance.

## 1. Introduction

Metals and alloys are widely used for various applications in industries because they possess good mechanical properties and are also inexpensive [1,2]. However, for all their good properties, they are subject to corrosion [3,4,5]. In fact, corrosion can be referred to as an insidious phenomenon because it behaves slowly and continuously and can cause major failures, although corrosion in some environments such as the sea may be very rapid and severe [6,7,8]. The economic losses caused by corrosion are always huge, although the environmental pollution caused by corroded materials is also of great importance [9,10]. Pollution control is always a priority, because it negatively affects people’s living environments and eventually reduces the well-being of residents and the physical health of people [11]. Over the years, researchers have investigated various materials that have multiple properties. Gels are one of these materials. According to their structure, various applications can be considered for them, such as lubrication, electronics, 3D printing, optical materials, tissue engineering, drug delivery, personal care, and the polymer industry [12,13]. For example, gels with spatially resolved structures can direct the stem cell fate to organ growth in vitro, or implantable gels can minimize abrasion-related inflammatory responses [14]. Hasanzadeh et al. [15] prepared 3D nanofibrous hydrogel scaffolds using fibrin, polyurethane, and multiwall carbon nanotubes. The results showed that the prepared scaffolds are able to support cell proliferation and also, that they are a suitable microenvironment for increasing cell viability. Shao et al. [16] prepared porous hydroxyapatite scaffolds using 3D gel-printing technology. The results showed that the prepared scaffolds have a pore size of about 350 µm × 350 µm and the maximum compressive strength and compressive modulus of the scaffolds are 16.77 and 492 MPa, respectively. Zhao et al. [17] prepared a polymer gel electrolyte, based on polyethylene glycol entrapped in a cross-linked cellulose structure. The results showed that the ionic conductivity was 3.31 × 10^−3^ Scm^−1^. Additionally, the assembled batteries by this electrolyte demonstrated initial discharge capacity of 159.3 mAh g^−1^ as well as the coulomb efficiency of 85.52% at 0.2 C.

Polymers are always one of the most amazing materials that are used in many fields. Polymer coatings are one of the ways to protect metals, in fact, they can be applied on different substrates [18,19,20,21,22]. The properties of polymer coatings, such as hydrophobicity, anti-fouling, self-healing, resistance to chemicals, and thermal, as well as their antimicrobial capabilities, have made them very popular [22,23,24]. The use of corrosion inhibitors and nanoparticles (such as carbon nanotubes, montmorillonite nanoparticles, and graphene oxide) can be very effective in increasing the lifetime of the coating [25,26,27,28]. Recently, green inhibitors, which are plant extracts and fruit waste, have received much attention because they are non-toxic, sustainable, and low-cost [29]. Although there are limitations when preparing plant extracts, such as the solvents used, the temperature of the preparation of the extract, and the temperature of drying the plant, all of which have an effect on the final quality of the product [30,31,32]. Dehghani et al. [33] used the extract of *Ziziphora* leaves as an inhibitor for the acidic-induced corrosion of mild steel. The results showed that by increasing the concentration of the extract, the inhibition performance increases to 93%. Barbouchi et al. [34] used the essential oils of twigs, leaves, and fruits of Terebinth (*Pistacia terebinthus* L.) as corrosion inhibitors for iron in the neutral chloride medium. The fruit essential oil, at a concentration of 3000 ppm, had the best anti-corrosion properties compared to other essential oils and showed an inhibition performance of 86.4%. Begum et al. [35] used extract from *Spilanthes acmella* aqueous leaves as an inhibitor for the acidic-induced corrosion of mild steel. The results showed that by increasing the concentration of *Spilanthes acmella*, the inhibition efficiency (IE) improves, but by increasing the temperature, the IE was reduced. Méndez et al. [36] used *Ilex paraguariensis* (Yerba Mate) as an inhibitor for the acidic-induced corrosion of aluminum. The results showed that by increasing the concentration of the extract, the inhibitory performance improves, and increasing the temperature has no effect on the inhibitory performance. Chapagain et al. [37] used the alkaloid of *Rhynchostylis retusa* as an inhibitor for the acidic-induced corrosion of mild steel. The results showed that the IE in gravimetric and polarization methods after 6 h of immersion is 87.51 and 93.24%, respectively. Additionally, this inhibitor can only work below 35 °C. Meanwhile, CT is a tropical flower, also known as butterfly pea, which is abundantly found in gardens and in the wild [38]. CT is native to Zimbabwe, Ghana, Guinea, Malaysia, and Indonesia but is also widely found in America, Australia, and South Africa [39,40]. CT is rich in bioactive compounds and has properties such as antidiabetic, antimicrobial, anticholesterol, anti-inflammatory, and antioxidant activities [41]. Hence, in many countries, CT is used in traditional and modern medicine, cosmetics, agriculture, and the food industry [42,43]. Sol-gel is known as an environmentally friendly synthesis method and is economically viable [44,45,46]. Briefly, it consists of three stages hydrolysis, polycondensation, and thermal densification [47]. The simple conditions of the sol-gel process (such as synthesis at room temperature and atmospheric pressure), high purity of the product, low toxicity, and cost-effectiveness are the advantages of this method [48,49,50].

**Figure 1:**
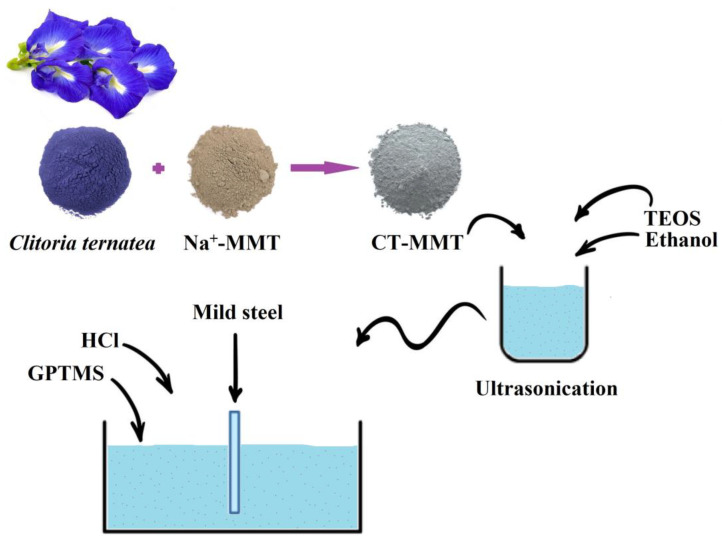
Schematic illustration of the preparation of coatings.

Herein, CT-MMT, tetraethyl orthosilicate (TEOS), and 3-Glycidyloxypropyltrimethoxysilane (GPTMS) have been used to prepare coatings with anti-corrosion and antimicrobial properties (see Figure 1).

## 2. Results and Discussion

### 2.1. Evaluation of the CT-MMT

**Figure 2:**
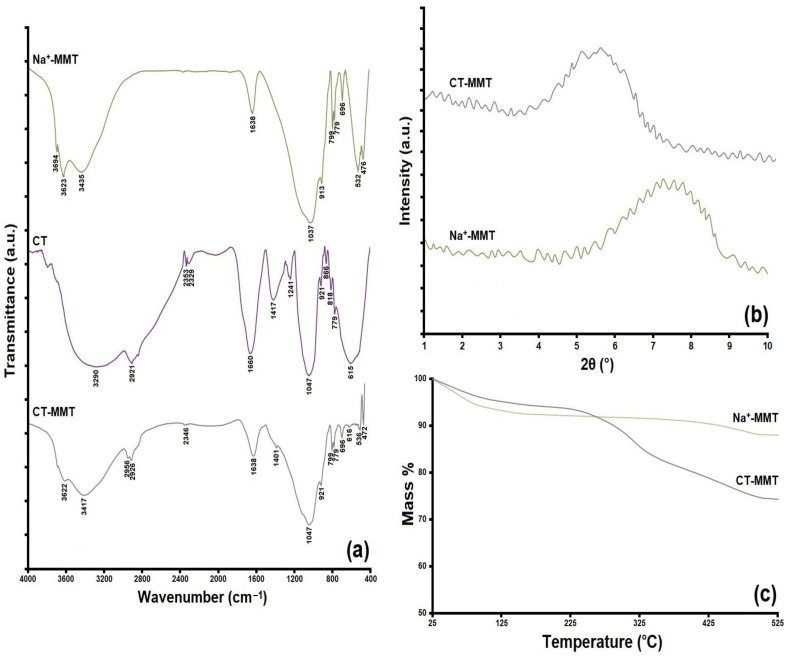
(**a**) FT-IR spectra and (**b**) XRD pattern (**c**) TGA curves of Na^+^-MMT, CT, and CT-MMT.

**Figure 3:**
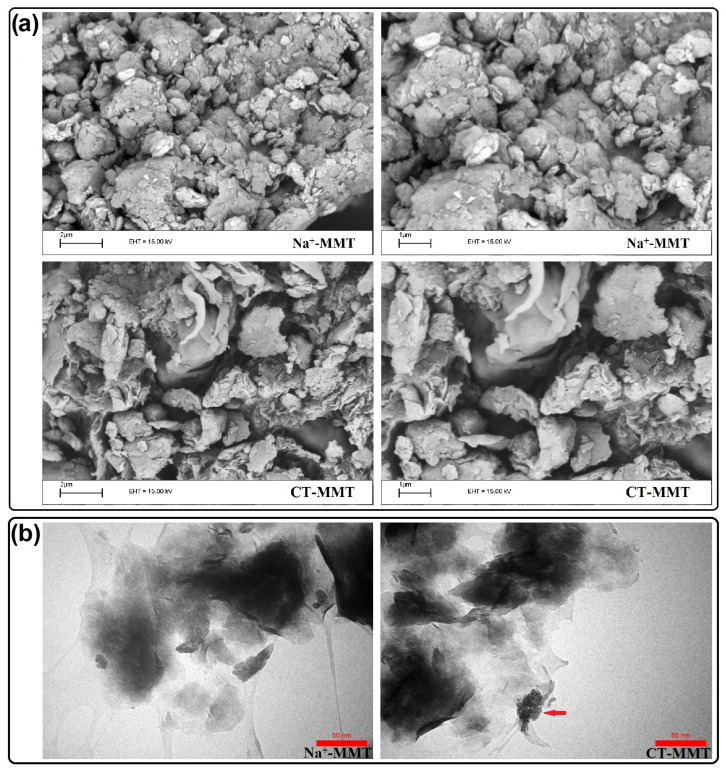
(**a**) SEM and (**b**) TEM images of samples Na^+^-MMT and CT-MMT.

Figure 2 and Figure 3 show the results for the CT-MMT modification investigation. As shown in the FT-IR results (in Na^+^-MMT spectrum), the peaks appearing at 476 and 1037 cm^−1^ are related to the bending and stretching Si-O vibrations [2,18]. Additionally, the peaks at around 532, 913, 1638–3435, and 3623–3694 cm^−1^ are corresponding to the Si-O-Al vibration and MgO groups, Al_2_OH bending groups, scissoring and symmetric vibrations of OH units, and stretching of OH (SiOH groups), respectively [2,18,51]. In the CT spectrum, the peaks appearing at 615–866 cm^−1^ are related to the primary and secondary amines [51]. The peaks at around 921, 1047, 1241, 1417 and 2921, 1660, 2329–2353, and 3290 cm^−1^ are corresponding to the OH bending vibration (carboxylic acids), aliphatic amines, alkyl halides, stretching vibrations of C=C groups, stretching vibrations of C-C groups, stretching vibrations of H-C=O groups, and OH stretching vibration (phenol), respectively [52,53]. In the CT-MMT spectrum, the peaks at around 616, 921, 1401, 2346, 2926, and 2956 cm^−1^ are corresponding to the presence of CT. Additionally, other bands observed in CT-MMT were related to the clay. The XRD pattern of the samples shows that they have peaks with a center at 2θ = 7.4° (d-spacing = 11.88 Å) and 2θ = 5.63° (d-spacing = 16.78 Å), respectively. It is observed that there is an increase in d-spacing in CT-MMT, which is due to the absence of Na^+^ cation and the presence of CT [2,18]. Figure 2c shows the TGA curves of Na^+^-MMT and CT-MMT. Na^+^-MMT in five stages of weight loss showed that the temperature range was between 25–100 (−5.95%), 100–150 (−1.48%), 150–350 (−1.13%), 350–415 (−0.84%), and 415–525 °C (−2.60%), respectively. However, the weight loss of CT-MMT occurs in the temperature ranges 25–100 (−4.29%), 100–230 (−2.34%), 230–330 (−8.58%), and 330-525 °C (−10.13%), respectively. The weight loss of Na^+^-MMT up to the range of 150 °C is related to dehydration inside the interlayer space, and in the rest of the ranges it is related to the dehydroxylation of inorganic clay [54,55]. In general, Na^+^-MMT had a 12% weight loss, but this value for CT-MMT was 25.34%. It can be said that the weight loss up to the range of 230 °C for CT-MMT is related to the loss of moisture and parts of CT that are on the surface of the clay. However, the weight loss in the rest of the temperature range is related to the content of CT between the clay plates. Finally, the temperature range of 25–100 °C can be considered to reduce moisture in both samples, in this case, Na^+^-MMT and CT-MMT had a 6% and 21% weight loss, respectively.

Figure 3 shows the SEM and TEM images of Na^+^-MMT and CT-MMT. SEM images show that the morphology changes partly after the exchange process. It seems that some of the plates are partly separated and the CT-MMT has greater disunion than the Na^+^-MMT. Although it has been reported in the literature that the morphology does not change significantly after modification [18,56]. Additionally, the TEM images (Figure 3b) show the presence of CT particles in MMT (indicated by the red arrow). According to the results, it can be said that the exchange of Na^+^ cations with CT has been successful.

### 2.2. Investigation of Anticorrosion Properties

**Figure 4:**
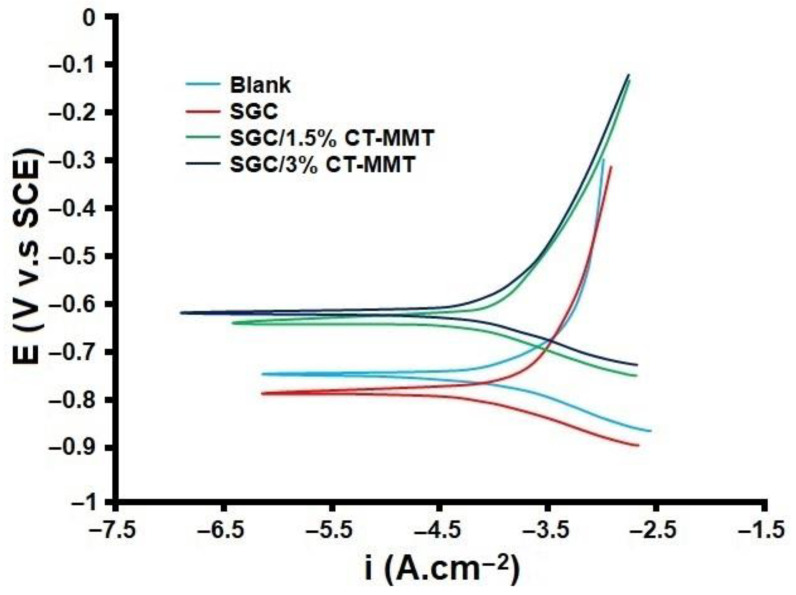
The polarization curves of samples immersed in 3.5 wt.% NaCl solution.

The electrochemical properties of the samples were investigated using the polarization test after 72 h of immersion in 3.5 wt.% NaCl solution (see Figure 4 and Table 1). The results show that applied coatings containing CT-MMT increase corrosion potential (E_corr_) values to a higher level and decrease corrosion current density (I_corr_), which indicates the creation of protective conditions for the substrate. As the results of Table 1 show, with the presence of CT-MMT in the matrix, the anodic Tafel slope (β_α_) and cathodic Tafel slope (β_c_) were affected, but the extent of this effect on the anodic half-reaction was more than the cathodic. Hence, CT-MMT can be placed in the group of anodic inhibitors, due to the overall displacement of E_corr_ by more than +85 mV [57]. As mentioned before, with the addition of CT-MMT to the matrix, I_corr_ has been greatly reduced, which indicates an increase in corrosion resistance. Additionally, the corrosion rate (C.R) for the samples containing CT-MMT is greatly reduced compared to the blank sample. The mild steel coated with pure sol-gel coating was able to significantly reduce I_corr_ and C.R due to its good barrier properties. Additionally, its E_corr_ shifted towards more negative values than the E_corr_ of the blank sample, which can be attributed to its cathodic performance mechanism. However, it is not suitable for long-term protection of the substrate, due to the presence of porosity.

**Figure 5:**
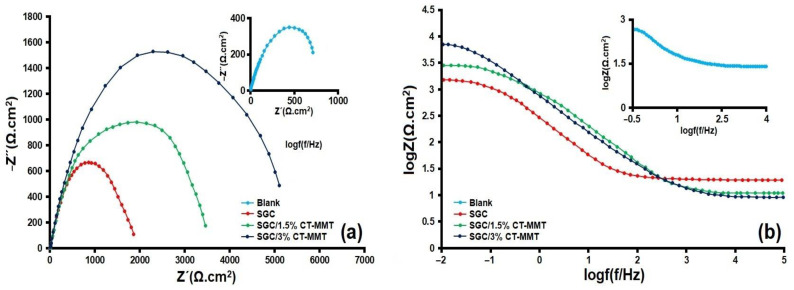
(**a**) Nyquist and (**b**) Bode diagrams of the samples after 72 h of immersion in 3.5 wt.% NaCl solution.

Figure 5 show the results obtained from the EIS test after 72 h of immersion in a 3.5 wt.% NaCl solution. According to Figure 5a (Nyquist diagrams), the diameter of the semicircle shows the charge transfer resistance (R_ct_), which expresses the corrosion rate. In fact, a semicircle diameter with a larger radius means a lower corrosion rate and the highest inhibition performance [58]. As the results show, the coatings containing 1.5 and 3 wt.% CT-MMT have the highest R_ct_ values after 72 h of immersion, while the lowest R_ct_ value is related to the blank sample, which can be attributed to the destruction of the oxide layer on the metal surface [59]. Bode diagrams (Figure 5b) clearly show that the corrosion resistance and impedance of the samples containing 1.5 and 3 wt.% CT-MMT are higher than the SGC and blank samples. Impedance values at low frequencies are related to the corrosion behavior at the interface of the coating and substrate, and impedance values at high frequencies are related to the performance of the coating in the electrolyte [60]. Therefore, the impedance for the sample containing 3% CT-MMT is higher than the rest of the samples in both high and low frequency, which indicates the great difference in its corrosion resistance. As can be seen, the samples covered by silane have two time constants. The first time constant is probably related to the silane layer, and the second time constant is related to the response of the interface. The curve related to the blank sample shows only a one-time constant that indicates the charge transfer process at the interface between the metal and the solution.

**Figure 6:**
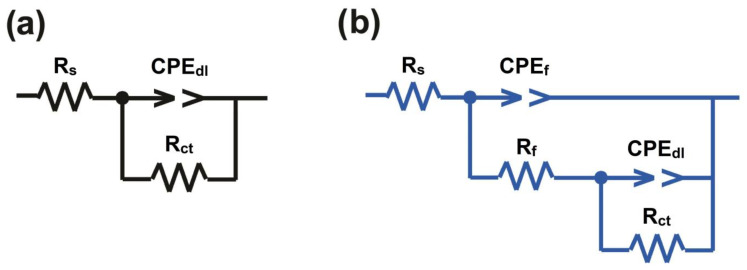
Equivalent circuits for the description of coatings: (**a**) one-time constant (**b**) two-time constant.

According to the results, two equivalent circuits were selected (see Figure 6). In Figure 6a, R_s_, R_ct_, and CPE_dl_ indicate solution resistance, charge transfer resistance, and the constant phase element related to the electrical double layer. Additionally, Figure 6b has R_f_ and CPE_f_, which show the resistance and the constant phase element related to the coating, respectively. Some parameters obtained from modeling EIS diagrams are presented in Table 2. In Table 2, n_dl_ and n_f_ represent the constant related to surface heterogeneity in the electrical double layer and coating, respectively. For the blank sample, the low value of n_dl_ compared to other samples indicates the occurrence of surface damage in the steel due to the invasion of chloride anion, which has caused the surface homogeneity to decrease [61]. The coating containing 3 wt.% CT-MMT has the highest amount of n_dl_, which indicates the increase in the uniformity of the sample. The SGC has the lowest n_f_ value, which is due to the presence of large porosity and rough surface in the coating [62]. Y_o,dl_ and Y_o,f_ indicate the admittance of the electrical double layer and the coating, respectively. The admittance of the samples shows that the samples containing CT-MMT prevent the access of corrosive agents to the active areas of the metal surface, by creating a compact film that is fortified with the plate structure of MMT and CT compounds in the matrix. For this reason, the value of Y_o,f_ has decreased. The decreasing trend of Y_o,dl_, in fact, shows the replacement behavior between water molecules by CT molecules that were released from the space inside the MMT layer (at the metal/solution interface) during the immersion times [63]. Additionally, the decrease in the capacitance of the electric double layer (C_dl_) is due to the decrease in the dielectric constant of the capacitor or the increase in the thickness of the capacitor, which indicates that fewer ions are placed on the surface of the metal, so fewer ions are exposed to the reaction, which indicates it forms an inhibitory film (See how to calculate C_dl_ in Section 4.4). On the other hand, the reduction of the R_f_ in the SGC is due to the penetration of water and corrosive ionic species into the matrix, which resulted in an increase in the conductivity of the coating. In fact, the presence of CT-MMT, with the ability to cover active sites on the surface of steel samples, was able to reduce the hydrophilicity of the coating (due to the hydrophobic compounds in CT) [41], which led to less electrolyte penetration and improved coating performance. According to the results, the sample containing 3 wt.% CT-MMT has the highest IE (See how to calculate IE in the Section 4.4).

**Figure 7:**
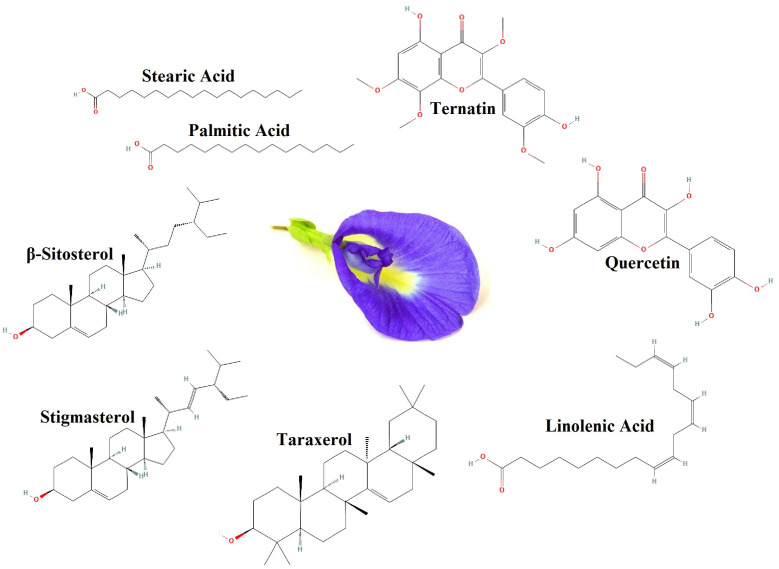
The most important anticorrosion compounds in CT.

CT contains various compounds such as ternatin, quercetin, linolenic acid, palmitic acid, taraxerol, *β*-sitosterol, stigmasterol, and stearic acid (see Figure 7) [41]. The oxygen groups in its compounds can form a protective layer on the surface by interacting with the anodic dissolution products (Fe^2+^) and blocking the anodic region. Additionally, due to the hydrophobic nature of CT compounds [41], hydrophilicity is reduced and the penetration of corrosive species is reduced. On the other hand, MMT has the ability to react with hydroxyl ions in the cathode region and causes the production of insoluble compounds, and prevents oxygen from reaching the cathode region [2,18,64].

### 2.3. Investigation of Antimicrobial Properties

**Figure 8:**
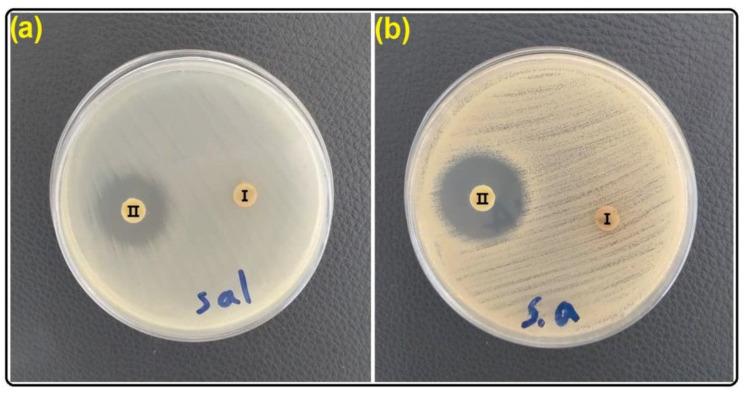
Antibacterial activity of (I) SGC and (II) SGC/3% CT-MMT: (**a**) *Salmonella paratyphi-A serotype* (**b**) *Staphylococcus aureus*.

**Figure 9:**
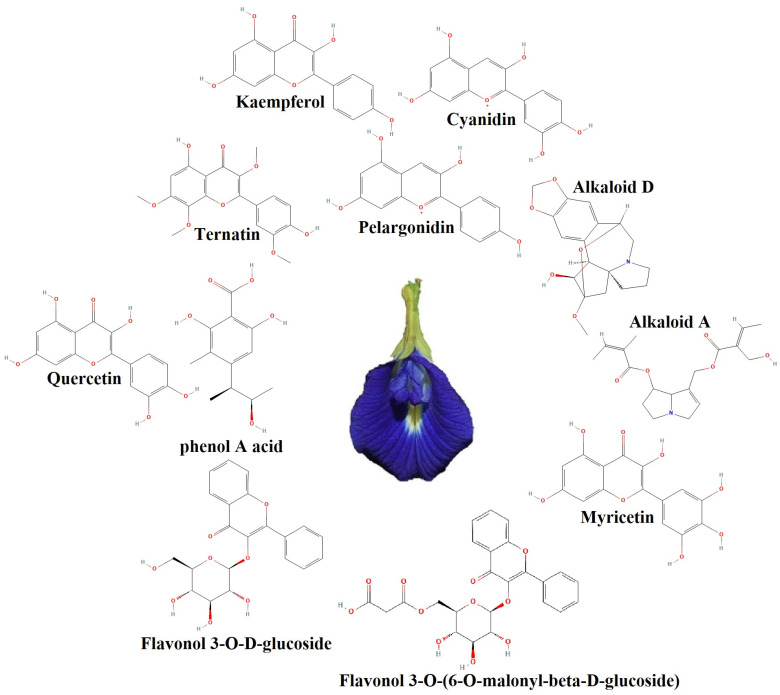
The most important antimicrobial compounds in CT.

Environments with high population density are prime candidates for cyclic transmission of viruses, bacteria, and fungi, such as hospital environments and subway stations [65]. Transmission of fungal and bacterial infections can occur through contact with surfaces. Antimicrobial coatings are definitely one of the most important achievements of human development that can be applied to surfaces and thus prevent the spread of disease [25,66]. Many factors affect pathogen deposition on the surface, such as hydrophilicity/hydrophobicity, roughness, charge, chemical composition, and temperature [67]. Usually, in the preparation of antimicrobial polymers, the focus is on long-range electrostatic interactions because it causes physical disturbances in the bacterial cell wall or membranes [25]. Researchers described five main antimicrobial mechanisms, including direct and indirect actions, pathogen receptor inactivation, photothermal effect, and antifouling behavior [67]. In fact, it can be said that in the preparation of coatings, if several properties are considered for them, the coatings will be much more practical. In this study, the antimicrobial properties of SGC and SGC/3% CT-MMT against two types of bacteria, (*Staphylococcus aureus* (gram-positive) and *Salmonella paratyphi-A serotype* (gram-negative)), were investigated (see Table 3 and Figure 8). As the results show SGC has no effect on bacteria. The formation of inhibition zones by SGC/3% CT-MMT is due to the presence of CT in the structure. CT is one of the most important sources of polyphenols [41]. CT contains cyanidin, pelargonidin, alkaloid A, alkaloid D, ternatin, kaempferol, phenol A acid, quercetin, myricetin, flavonol 3-O-D-glucoside, and flavonol 3-O-(6-O-malonyl-beta-D-glucoside). That is why it has immune-enhancing, anti-inflammatory, anti-cancer, antioxidant, and antibacterial effects (see Figure 9) [41,42,43]. Phenolic compounds have the ability to suppress by membrane perturbation, reduction of host ligands adhesion, and neutralizing bacterial toxins [68]. Usually, polyphenols and anthocyanins of edible plants are used as functional ingredients in beverages because they have the ability to prevent different diseases [41]. The results show the greater effect of CT on gram-positive bacteria, which is due to the difference in the structure of the cell-wall (gram-positive and gram-negative bacteria). In fact, the cell-wall of gram-positive bacteria is simple and porous, so it is easier to penetrate, but the structure of the cell-wall of gram-negative bacteria is complex and less porous [25].

## 3. Conclusions

To sum up, we successfully added CT to the Na^+^-MMT structure. After modification of Na^+^-MMT, 2θ changes, and the *d*-spacing increases, which was owing to the presence of CT in the interlayer space of MMT. Additionally, the presence of CT-MMT increased the corrosion resistance and, by the increase of its content, the corrosion resistance increased. The inhibition efficiency for the sample containing 3 wt.% CT-MMT was 86%, while this value was 61.7% for the pure coating. Moreover, the antimicrobial study showed that CT-MMT can have an inhibitory effect on *Staphylococcus aureus* and *Salmonella paratyphi-A serotype* bacteria. Considering the properties of CT, it can be said that the use of plants and their extracts can be effectively useful in creating and improving many properties such as anticorrosion and antimicrobial. On the other hand, their use does not harm the environment.

## 4. Materials and Methods

### 4.1. Materials

Na^+^-MMT and CT were obtained from Rockwood Company (Washington, DC, USA) and Peptina Company (Tehran, Iran), respectively. TEOS, GPTMS, hydrochloric acid (HCl), and ethanol were procured from Sigma-Aldrich Company (Schnelldorf, Germany). Mild steel was used for the substrates (5 cm × 10 cm), which was obtained from Mobarakeh Iranian Steel Company (Mobarakeh, Iran). The substrates were polished with corundum papers (600 to 1200 grades) and then cleaned with acetone.

### 4.2. Modification of Na^+^-MMT by CT

Briefly, Na^+^-MMT (20 g) was added to distilled water (2000 mL, room temperature) and stirred for 24 h. CT powder (8 g) was added to distilled water (800 mL, room temperature) and stirred for 2 h, and then added to the previous mixture. Then, the mixture was stirred (24 h at room temperature) and next the mixture was allowed to rest for 24 h. Finally, the mixture was centrifuged (20 min at 6000 rpm) and the final product (CT-MMT) was obtained and vacuum-dried at 30 °C for 36 h.

### 4.3. Preparation of Coatings

The coatings were synthesized by combining GPTMS and TEOS. For this purpose, GPTMS (7.5 mL) and TEOS (22.5 mL) were added to a beaker and then ethanol (10 mL) was added to the mixture. Finally, HCl (1 mL) as a catalyst was added dropwise to the mixture. To prepare nanocomposites; TEOS was first added to ethanol, and then CT-MMT was added to them and dispersed by ultra-sonication. Finally, GPTMS and then HCl were added to the mixture. The CT-MMT content in the samples was 1.5 and 3 wt.%. To assemble the coating on the surface of mild steel, the immersion method was used (dipping upright for 24 h). Then, the samples were placed in a laminar flow cabinet (24 h, room temperature).The thickness of all coatings was 5 μm.

### 4.4. The Measurements and Characterization

FT-IR spectra of the samples were recorded on an Equinox 55 spectrometer (Bruker, Mannheim, Germany). XRD measurements were carried out using an Xpert Pro MPD diffractometer (Panalytical, Almelo, The Netherlands). SEM equipment was ZEISS LEO-1430 VP (Carl Zeiss AG, Jena, Germany). The TEM was a Philips CM-120 (Amsterdam, The Netherlands). Antibacterial properties were investigated through the agar diffusion method as described in the literature [25], and the MIC and MBC were also investigated. The film thickness was investigated by Elcometer A456CFBI1 (Manchester, UK, 0–1500 μm, Ferrous). For electrochemical tests, after coating and curing, the samples were completely covered with a mixture of Beeswax melt and colophony resin, and only a 1 cm^2^ area of the surface of the coated steel sample was exposed to the 3.5 wt.% NaCl solution. EIS and polarization tests were performed by a three-electrode system including a calomel-type reference electrode (SCE), graphite-type auxiliary electrode, and working electrode (coated sample). These analyses were performed using the Corrtest CS350 potentiostat-galvanostat (Wuhan, China). Impedance for all samples was obtained after 72 h of immersion in the electrolyte at 25 ± 3 °C. Additionally, in the open circuit potential (OCP), an applied sine amplitude with a voltage range of 10 mV was used in the frequency range of 10 kHz to 10 mHz. Data processing was performed using Zview software3.1c. C_dl_ and IE were calculated by Equations (1) and (2), respectively [69].

(1) $$C_{dl}=Y_{o,dl}{}^{{}^{\frac{1}{n}}}{\left(\frac{R_{s}{}^{}R_{ct}}{R_{s}{+}^{}R_{ct}}\right)}^{\frac{1-n}{n}}$$

(2) $$IE(\%)=(\frac{{}^{}R_{ct{,}_{}sol-gel(CT-MMT)}-R_{ct,}{}_{blank}}{R_{ct{,}^{}sol-gel(CT-MMT)}})\times 100$$

In Equation (2), R_ct,sol−get(CT−MMT)_ and R_ct,blank_ respectively indicate the charge transfer resistance of sol-gel coatings containing different contents of CT-MMT and blank sample. The polarization test was performed with a scan rate of 1 mV/s in the potential range of −250 to +250 mV compared to the OCP. I_corr_ values were calculated using the linear region of the curves (±50 mV potential) through Tafel extrapolation. The C.R was also calculated by Equation (3).

(3) $$C.R=\frac{0.0032^{}I_{corr^{}}MW}{nd_{}}$$

In the mentioned equation, corrosion current density (μA·cm^−2^), molar mass (g·mol^−1^), charge number, and density (g·cm^−3^) of the tested metal are I_corr_, MW, n, and d, respectively.

## Figures and Tables

**Table 1:** Electrochemical parameters obtained from the polarization test on MS samples with and without sol-gel coating.

Sample	I_corr_ (μA·cm^−2^)	E_corr_ (mV/SCE )	β_α_ (mV·dec^−1^)	β_c_ (mV·dec^−1^)	C.R (mm·y^−1^)
**Blank**	−314.13	−741.68	187.24	367.48	98.54
**SGC**	−61.24	−786.41	96.87	60.38	23.70
**SGC/1.5% CT-MMT**	−31.59	−651.34	58.69	43.10	9.43
**SGC/3% CT-MMT**	−17.19	−629.22	43.61	33.64	6.25

**Table 2:** Electrochemical parameters obtained from EIS results for samples after 72 h of immersion in 3.5 wt.% NaCl solution.

Sample	R_S_ (Ω·cm^2^)	R_ct_ (Ω·cm^2^)	CPE_dl_ Y_o,dl_ (mΩ^−1^·cm^−2^) n_dl_		C_dl_ (μF·cm^2^)	CPE_f_ Y_o,f_ (mΩ^−1^·cm^−2^) n_f_		R_f_ (Ω·cm^2^)	IE (%)	log |z| (Ω·cm^2^)
**Blank**	27.41	715	0.82	0.71	879	-	-	-	-	2.6
**SGC**	23.87	1866	0.65	0.77	845	0.94	0.67	218	61.7	3.1
**SGC/1.5% CT-MMT**	25.34	3459	0.38	0.79	636	0.62	0.69	359	79.3	3.4
**SGC/3% CT-MMT**	27.79	5106	0.09	0.86	95	0.28	0.85	687	86	3.8

**Table 3:** Antimicrobial activity of samples.

** MBC	** MIC	* DD	Microorganism
- ^a^	- ^a^	- ^a^	***Staphylococcus aureus* (ATCC 29737)**
1000 ^b^	500 ^b^	9 ^b^	
- ^a^	- ^a^	- ^a^	***Salmonella paratyphi-A serotype* (ATCC 5702)**
2000 ^b^	1000 ^b^	6 ^b^	

Note: * DD: Disk diffusion method, inhibition zones in diameter (mm) around the impregnated disk. DD: 600 μg per well. ** MIC: Minimum inhibitory concentration, MBC: Minimum bactericidal concentration. Concentrations of MIC and MBC as μg/mL. ^a^ SGC, ^b^ SGC/3% CT-MMT.

## Data Availability

The datasets generated and/or analyzed during the current study are not publicly available at this time as the data form part of an ongoing study. However, the datasets are available from the corresponding author (Milad Sheydaei, mi_sheydaei@sut.ac.ir) on reasonable request.
